# Detection of potentially valuable polymorphisms in four group I intron insertion sites at the 3'-end of the LSU rDNA genes in biocontrol isolates of *Metarhizium anisopliae*

**DOI:** 10.1186/1471-2180-6-77

**Published:** 2006-09-15

**Authors:** Marcela Márquez, Enrique A Iturriaga, Enrique Quesada-Moraga, Cándido Santiago-Álvarez, Enrique Monte, Rosa Hermosa

**Affiliations:** 1 Centro Hispano-Luso de Investigaciones Agrarias (CIALE), Departamento de Microbiología y Genética, Universidad de Salamanca, Edificio Departamental lab 208, Plaza Doctores de la Reina s/n, 37007 Salamanca, Spain; 2 Area de Genética. Departamento de Microbiología y Genética, Universidad de Salamanca, Edificio Departamental lab 324, Plaza Doctores de la Reina s/n, 37007 Salamanca, Spain; 3 Departamento de Ciencias y Recursos Agrícolas y Forestales, Universidad de Córdoba, Edificio C4 Celestino Mutis, Campus Rabanales, 14071 Córdoba, Spain

## Abstract

**Background:**

The entomopathogenic anamorphic fungus *Metarhizum anisopliae *is currently used as a biocontrol agent (BCA) of insects. In the present work, we analyzed the sequence data obtained from group I introns in the large subunit (LSU) of rDNA genes with a view to determining the genetic diversity present in an autochthonous collection of twenty-six *M. anisopliae *isolates selected as BCAs.

**Results:**

DNA fragments corresponding to the 3'-end of the nuclear LSU rDNA genes of 26 *M. anisopliae *isolates were amplified by PCR. The amplicon sizes ranged from 0.8 to 3.4-kb. Four intron insertion sites, according to *Escherichia coli *J01695 numbering, were detected- Ec1921, Ec2066, Ec2449 and Ec2563- after sequencing and analysis of the PCR products. The presence/absence of introns allowed the 26 isolates to be distributed into seven genotypes. Nine of the isolates tested showed no introns, 4 had only one, 3 two, and 10 displayed three introns. The most frequent insertion sites were Ec1921 and Ec2449. Of the 26 isolates, 11 showed insertions at Ec2563 and a 1754-bp sequence was observed in ten of them. The most-parsimonious (MP) tree obtained from parsimony analysis of the introns revealed a main set containing four-groups that corresponded to the four insertion sites.

**Conclusion:**

Four insertion sites of group I introns in the LSU rDNA genes allowed the establishment of seven genotypes among the twenty-six biocontrol isolates of *M. anisopliae*. Intron insertions at the Ec2563 site were observed for first time in this species.

## Background

The use of entomopathogenic microorganisms to combat insects is currently considered to be a viable supplement or alternative to synthetic chemical insecticides, which are known to have toxic effects on non-target organisms [1]. Strains of *Metarhizium *have been shown to efficiently control several insect species [2,3]. The most common species is *M. anisopliae*, initially described as a pathogen of the wheat cockchafer *Anisoplia austriaca*. This fungus is commonly known as "green muscardine fungus" [4] and can infect the larvae and adults of more than 200 host species [5,3]. Once *M. anisopliae *spores have come into contact with the outer surface of the insect, they germinate. After penetrating the insect exoskeleton, they rapidly grow inside the insect and cause its death.

Traditionally, the classification and typing of anamorphic entomopathogenic fungi have mainly been based on morphological characteristics [6]. Nevertheless, such tools are insufficient for distinguishing between species of *Metarhizium *[7] or for monitoring the establishment and spread of a given strain released into the field, since these characters are modified by environmental and physiological conditions [8].

The application of molecular techniques in mycology has shed new light on the systematics, biochemistry, and ecology of entomopathogenic fungi [9]. Molecular markers have been used to assess the genetic variation among isolates of *M. anisopliae *and other entomopathogenic fungi in order to identify strains of interest, determine the origin of the isolates, study populations, or carry out phylogenetic analyses. Thus, a useful polymorphism for fingerprinting *M. anisopliae *isolates was detected using restriction fragment length polymorphism (RFLP) analysis of mitochondrial (mt) DNA [10]. A high degree of polymorphism was also detected in coding regions of small and large subunits of nuclear ribosomal RNA genes (SSU rDNA and LSU rDNA) as well as in intergenic spacers (IGS), whereas the internal transcribed spacers (ITS) were extremely conserved among the *M. anisopliae *isolates tested [11,12]. This rDNA polymorphism has been attributed to small insertions/deletions, multiple duplications, or -mainly- to the presence of group I introns [11].

Group I introns are autonomous genetic elements characterized by their ability to "self-splice", or to splice due to their particular topology. These introns are found in eukaryotic and (eu)bacterial domains [13,14]. In entomopathogenic fungi, the first report of a group I intron was described by Neuvéglise and Brygoo [15] for *Beauveria brongniartii*. RFLP analysis of PCR products revealed the presence of insertional elements of about 350–450 bp within the LSU rDNA. Several authors have reported the usefulness of group I introns, inserted after specific sites in SSU or LSU rDNA genes for genotyping species and strains in genera such as *Beauveria *or *Cordyceps *[16-20]. The presence of group I introns in *M. anisopliae *has been identified at three different positions within the LSU rDNA [11,12,17].

The present study was undertaken to determine any genetic diversity existing in an autochthonous collection of *M. anisopliae *isolates selected as biocontrol agents against insects. Twenty-six biocontrol isolates, most of them obtained from different locations in the Iberian Peninsula, were examined, based on the analysis of sequence data from group I introns in the LSU rDNA genes. The usefulness of group I introns in LSU rDNA genes for the characterization and identification of *M. anisopliae *strains was evaluated.

## Results

### Identification of group I introns

DNA fragments from LSU rDNA genes of 26 *M. anisopliae *isolates (Table 1) were amplified with primers I29/E24. The PCR products were purified and sequenced with a view to identifying whether multiple insertion sequences might be responsible for the length diversity in this region. The intron-less LSU sequence size was 823 bp. Sequence analysis revealed that nine isolates -designated Ma4 Ma7, Ma13, Ma18, Ma19, Ma20, Ma23, Ma24 and Ma25- had no introns. In the other seventeen isolates, the presence of introns inserted at one or more of the four possible conserved positions was observed. Intron insertion sites were refered to the I29 primer and located after positions 73 (Ec1921), 217 (Ec2066), 625 (Ec2449) and 738 (Ec2563). These insertion sites were named according to numbering of their nucleotide position in *Escherichia coli *J01695. Inserted intron sequence sizes were 440-bp at Ec1921; 343 bp at Ec2066; 409, 413, 414 or 417 bp at Ec2449; and 435 or 1754 bp at Ec2563.

Among the 17 intron-containing isolates, ten of them showed a length of 3.4-kb with three introns inserted at positions Ec1921, Ec2449 and Ec2563. One isolate was 1674 bp in length, with two introns at positions Ec2449 and Ec2563. Another two were 1687 bp long, with two introns at positions Ec1921 and Ec2449. Another isolate was 1367 bp long, with an intron at position Ec2449. Only one isolate, with an amplicon of 1173 bp long, exhibited an intron at position Ec2066. Two isolates from Cantabria (North Spain) were 1270 bp long, with one intron at position Ec1921.

The presence/absence of introns at the 3'-end of the nuclear LSU rDNA of the twenty-six *M. anisopliae *isolates analyzed allowed a distribution into the following genotypes: A1B2A3A4, B1B2A3A4, A1B2A3B4, B1B2A3B4, B1A2B3B4, A1B2B3B4 and B1B2B3B4. Letters A and B indicate the presence and absence of introns, respectively, and the insertion sites are numbered from 1 to 4; that is, Ec1921 (1), Ec2066 (2), Ec2449 (3) and Ec2563 (4). These genotypes and their distribution frequencies are shown in Table 2. Nine (34.6%) out of the twenty-six strains had no introns; four (15.4%) contained one; three (11.5%) had two, and ten (38.5%) had three introns. The most frequent insertion sites -53.8% of the isolates studied- occurred at nucleotide positions Ec1921 and Ec2449, followed by position Ec2563 (42.3%), whereas only one strain had an intron at Ec2066 (3.8%).

### Intron characterization and variability

A 440-bp intron was identified in fourteen isolates at position Ec1921. Alignment of these sequences revealed the existence of two variants with a few bp differences for this intron. The divergences were due to nucleotide substitution at different positions. BLAST analysis of these sequences revealed high sequence identity with an intron inserted at the same position in the LSU from *M. anisopliae *33 (97% and 98%, respectively; AF197122) [11] and with another intron also inserted at the same position in the LSU from *M. anisopliae *IMNST 9601 (96 and 95% identity; AF363479).

Isolate Ma17 from Seville was the only one of the twenty-six isolates selected for study that showed evidence of an intron, 343 bp long, at position Ec2066. This intron had 95 and 98 % identity with two sequences previously detected at the same position in the LSU of *M. anisopliae *isolates 11 and 33 (AF197121 and AF197123, respectively).

Fourteen isolates displayed one intron at position Ec2449. Five sequence variants, ranging from 409 to 417 bp in size, were identified at this site. These five sequences were very similar and showed a 96–98 % identity with the Ec2449-position intron sequence reported for *M. anisopliae *33 (AF197124).

Two sequence types, very different in length, were detected at position Ec2563 of eleven of the *M. anisopliae *isolates. One of them was 1754-bp in size sequence and was detected in ten of these isolates. A 161-bp fragment at the 3' end of this sequence showed a BLASTn identity of 83% with a *Cordyceps *intron sequence from position 1398 to 1585 (AB044641). The rest of this 1754-bp sequence did not show either BLASTn or BLASTx identity with any of the genome sequences deposited in the public databases. An open reading frame (ORF) coding for 360 amino acids, located at position 49-1084-bp, was identified in this sequence. No homing endonuclease (HE) motifs were detected. The second type was observed in only one isolate, Ma12, corresponding to a 435-bp long intron showing a sequence of 172-bp with a 84% of identity, from position 125 to 297, with the same intron sequence of *Cordyceps *(AB044641).

In order to explore the different intron sequences obtained in the present work in greater detail, a comparative study with elements P, Q, R and S, previously reported in group I introns [13], was carried out (a. n. AF197122 for Ec1921 site; a. n. AF197123 for Ec2066 and a. n. AF197124 for Ec2449). Since the Ec2563 insertion site had not been described previously in *M. anisopliae*, the comparison was performed with the *B. bassiana *sequence a. n. AF430699. P, Q and R elements were conserved at the four insertion sites. However, the element S was also identical in introns located at the Ec1921, Ec2066 and Ec2563 sites, but was not conserved for introns inserted at position Ec2449. At Ec2563 site the four elements were located in the 1754-bp intron at the following positions: 1428 to 1440 (P), 1505 to 1519 (Q), 1455 to 1466 (R) and 1553 to 1564 (S). In the 435-bp intron, these elements were located at positions 141 to 153 (P), 168 to 179 (Q), 218 to 232 (R) and 265 to 276 (S).

### Phylogenetic analysis

**Figure 1:**
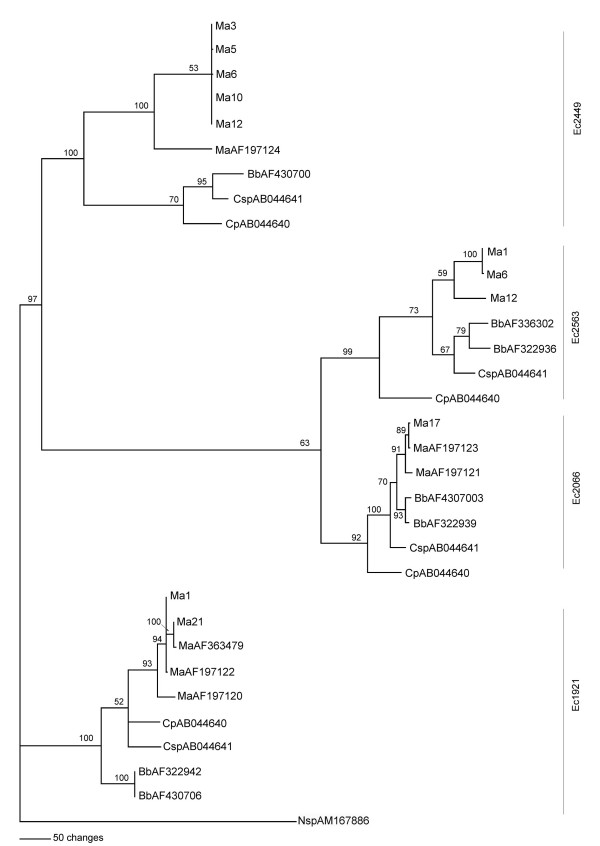
**Phylogenetic analysis of group I introns of the LSU rDNA genes from entomopathogenic fungi. **The MP tree was generated by parsimony analysis after heuristic searching (TBR option). Bootstrap full heuristic analysis with bootstrap intervals from 1000 replications and nodes supported in >50% of bootstrap replicates was generated using the PAUP 4.0 program. The branch lengths are proportional to the number of changes. Eleven different intron sequence types identified from 26 *Metarhizium anisopliae *isolates were aligned with 21 representative intron sequences from *Metarhizium anisopliae *(Ma), *Beauveria bassiana *(Bb), *Cordyceps profilica *(Cp) and *Cordyceps *sp. (Csp) and, and an intron sequence from *Naegleria *sp. (Nsp) was used as an outgroup.

The MP tree obtained after the alignment of 11 distinct intron sequence types identified from 26 isolates and GenBank-deposited sequences, that represent intron sequences from three entomopathogenic genera *Metarhizium, Beauveria *and *Cordyceps*, and the subsequent phylogenetic analysis is shown in Figure 1. The MP tree shows the separation of four independent groups corresponding to introns located at positions Ec1921, Ec2066, Ec2449 and Ec2563, supported by high bootstrap values (100, 92, 100 and 99%, respectively). Clusters grouping intron sequences located at Ec2066 and Ec2563 were closer although with a support of 63 %.

## Discussion

The variability in group I introns from LSU rDNA genes can be used as a robust molecular tool for the identification of polymorphisms in entomopathogenic fungi such as *Beauveria *and *Metarhizium *[11,16,21]. The primers previously described to amplify the 3'-end of the LSU rDNA region in *Beauveria *by PCR were used to characterize our *M. anisopliae *isolates. The polymorphism observed in this region of *M. anisopliae *genome was high, due to the presence/absence of introns at the four conserved sites. In the 26 isolates of *M. anisopliae*, we identified a total of 40 group I introns, which were inserted at any of the four specific sites in the LSU rDNA. This degree of polymorphism is in agreement with the high number of group I introns detected in *Cordyceps*, in which 28 representative members of this genus have been studied [18]. The latter authors suggested that if sequence comparisons are carried out between closely related taxa, one would expect to find a high variety of insertion patterns. Thus, considering the presence/absence of introns, due to intron vertical transmission and intron loss events, it was not unexpected that we found six genotypes when we analysed the 26 isolates belonging to the same species. The eight insertion sites described in rDNA genes of entomopathogenic fungi are not always occupied by introns and this could be related to the genus or species considered. When LSU introns were explored, 47 isolates of *B. brongniartii *and 125 of *B. bassiana *were distributed within 9 and 13 genotypes, respectively. A high number of introns inserted at the four specific sites was detected for both species, and only 7% of isolates tested did not display intron insertions [16,20]. However, in other studies of *M. anisopliae *a low intron number was detected; only 5 and 2 introns were described from 30 and 40 strains studied, respectively [11,12]. Bearing in mind the taxa sampled, those *M. anisopliae *results were surprising since a high degree of polymorphism, due to vertical transmission events, would be expected, and an even higher degree if the high polymorphism discovered in the rDNA genes of *Cordyceps*, the teleomorph of these species, was considered [21,22].

Phylogenetic studies have been used to explain the sporadic distribution of these elements in *Cordyceps*, proposing a cyclic model of invasion, degeneration, loss, and reinvasion [18]. However, genotypes 1 (three introns) and 7 (no-intron insertions) were predominant in our study, with a frequency of 38.5% and 34.6 %, respectively. Moreover, introns were more frequent at positions Ec1921 and Ec2449 (53.8%). Additionally, only one intron inserted at Ec2066 was discovered (3.8%). In our hands, and light of these distribution frequencies, it seems that there is some kind of preference for certain positions. LSU sites where intron insertion is more frequent (84.4% for Ec1921) have also been detected in *B. bassiana *strains and isolates with three-, two- and one-intron insertions, representing 16%, 48% and 28% frequency, respectively [20].

A phylogenetic analysis based on intron sequences in the LSU rDNA was carried out with the 11 different *M. anisopliae *sequences obtained in this work and some other *Metarhizium*, *Beauveria *and *Cordyceps *representative sequences from public databases. In general, the MP tree shows that introns inserted at the same site are closely related. This is expected, considering that horizontal transmission is one of the pathways along which group I introns can move [18,23]. This is also in agreement with the results obtained in other entomopathogenic fungi, in which phylogenetic analyses of group I introns suggest that transposition between different insertion sites is uncommon [12,20]. At the Ec2449 insertion site, *M. anisopliae *isolates are separated in an independent clade (bootstrap of 53%) from the *Beauveria *and *Cordyceps *isolates. In spite of the sequence polymorphism observed at this insertion site, the five variants of *M. anisopliae *grouped together. In this sense, a sequence comparison of the P, Q, R and S motifs in introns inserted at the four specific sites was carried out (data not shown). Compared with AF197124 sequence of *M. anisopliae*, only P, Q and R motifs were conserved.

An intron at insertion point Ec2563 was identified in eleven isolates. Nevertheless, the intron of the ten isolates grouped in genotype 1 had an uncommon size of 1754-bp. This intron type is usually small, ranging in size from 250–500 bp [24]. Introns with similar lengths containing an open reading frame (ORF) encoding a highly specific homing endonuclease (HE) have been reported previously in both the SSU of *B. bassiana *[25] and the LSU rDNA of *Naegleria *sp. [26]. HE conserved motifs were not detected in the 360 amino acid sequence of the *M. anisopliae *ORF, suggesting the ORF is not an HE.

Species and strains of *Metarhizium *and *Beauveria *have been differentiated by RAPD analysis, but little evidence of a correlation between RAPD groups and the geographical origin or host has been gathered [27]. Other studies carried out in *M. anisopliae *have described that similar RAPD profiles tend to be obtained from isolates of the same geographic area [28]. LSU introns have shown a tenuous dependence upon geographical origins or specific insects with rDNA intron genotype distribution in 125 strains of *B. bassiana *[20]. In the present study, the number of isolates was much lower and those obtained from insects were only two. Then, no comparisons between genotypes and habitat/host could be made. In the present work we failed to observe any correlation between the distribution of genotypes and the geographical origins of the isolates.

The genus *Metarhizium *includes entomopathogenic isolates used as BCAs. To predictably and successfully apply BCAs to combat insects in the field, it is essential that their biology and ecology should be well understood. The release of biocontrol agents into the environment demands the development of methods to monitor their presence or absence in the field [29,30]. Using a molecular marker such as an LSU rDNA intron, two *B. bassiana *strains were discriminated in co-formulation studies [31]. In this study, the twenty-six isolates analyzed were distributed into seven genotypes considering the presence/absence of group I introns at the 3'-end of the LSU rDNA genes. Thus, three of the seven genotypes established (genotypes 2, 4 and 5) have a single component; and a high degree of polymorphism was also observed in the S motif of intron sequences inserted at Ec2429. Primers have been designed in these genes and their specificity is currently being tested with both mixed mycelia from different *M. anisopliae *strains and soil samples with a view to generating SCAR markers and developing quantitative real-time PCR assays.

## Conclusion

A total of 40 group I introns related to four insertion sites detected at the 3' end in LSU rDNA genes have been identified in 26 biocontrol isolates of *M. anisopliae*. These introns exhibited various insertion patterns and this small and selected collection of 26 isolates was distributed into 7 genotypes. Phylogenetic analysis of these intron sequences demonstrated a general correlation between specific insertion sites and sequence homology. P, Q, R and S elements were conserved at insertion positions Ec1921, Ec2066 and Ec2563, and only P, Q and R elements were conserved at Ec2449 site.

## Methods

### Fungal strains and culture conditions

Twenty-six strains of *M. anisopliae *were used in this study. The cultures were obtained from the collection at the CRAF (Ciencias y Recursos Agrícolas y Forestales) Department of the University of Cordoba (Córdoba, Spain). Their habitat/host and geographical origins are shown in Table 1. Cultures were maintained on malt extract agar (MEA, Difco Becton Dickinson, Sparks, MD) plates at 4°C.

### Genomic DNA extraction

Mycelia for DNA extraction were obtained by inoculating MEA plates that contained a sterile cellophane sheet with spores from MEA cultures after incubation at 25°C for 4 days. Mycelia were collected by scraping the cellophane sheets and were then frozen, lyophilized and ground. Total DNA was extracted using the method described by Möller *et al. *[32].

### PCR amplification and sequencing

The primers used for the amplification of the 3'-end of the LSU rDNA region have been reported previously for the amplification of this region in the genus *Beauveria *[16,20]. Amplification of this rDNA region for the 26 isolates of *M. anisopliae *included in this study was carried out with the primer pair E24 (5'- GCTGAATTACCATTGCGGAG-3') and I29 (5'-CTGCCCAGTGCTCTGAATGTC-3'), using the *Taq polymerase *system (Biotools B&M Labs, Madrid, Spain), following the manufacturer's instructions. PCR was performed in a total volume of 50 μl containing 25 ng of genomic DNA and 0.20 μM concentrations of each primer described above. The amplification included an initial denaturing cycle of 5 min at 94°C, followed by 35 cycles of 1 min 30 sec at 94°C, 2 min 30 sec at 57°C and 3 min at 72°C, and a final extension step of 5 min at 72°C in a PCR System 9700 Genetic Thermal Cycler (Applied Biosystems, Foster City, CA). The PCR products were electrophoresed on 1% agarose gels buffered with 1X TAE [33] and stained with ethidium bromide. A 100-bp ladder molecular weight standard (Roche Mannheim, Germany) was also used.

The PCR products were purified from agarose gels using the GenClean II kit (Q-BioGen, Carlsbad, CA) according to the manufacturer's protocol. DNA sequences were obtained using an automated ABI 377 Prism Sequencer (Applied Biosystems) with fluorescent terminators. All PCR products were sequenced in both directions. Sequences of internal primers were as follows: M1 (LSU gene, position 769–790), 5'-GGTAAAACTAACCTGTCTCACG-3'; M2 (1754 bp intron, position 184–202), 5'-GATCCTTCCCAGGGAGGTG-3'; M3 (1754 bp intron, position 1238–1257), 5'-CGGGTAGGTATTGTAAGGCG-3'; M4 (1754 bp intron, position 542–559), 5'-GGGACCTCGAGGTTTGTG-3' and M5 (1754 bp intron, position 893–911), 5'-CCAGGTCTGACTTACATG-3'.

### Molecular analysis

The presence or absence of introns at the 3'-end of the nuclear LSU rDNA of *M. anisopliae *was analyzed by detecting previously described target sequences [20]. Sequence alignments were generated using the MegAlign (DNASTAR package, 1989-92, London, UK) and the CLUSTALX 1.81 program [34]. Published intron sequences for isolates included within the genera *Metarhizium*, *Beauveria*, *Cordyceps *or *Naegleria *were retrieved from GenBank and included in the alignments. The GenBank accession numbers were: *B. bassiana*, AF322942 and AF430706 for insertion site Ec1921; AF430703 and AF322939 for insertion site Ec2066; AF430700 for insertion site Ec2449, and AF336302 and AF322936 for insertion site Ec2563; *M. anisopliae*, AF197120, AF197122 and AF363479 for insertion site Ec1921; AF197121 and AF197123 for insertion site Ec2066, and AF197124 for insertion site Ec2449, and *Cordyceps prolifica*, AB044640, and *Cordyceps *sp., AB044641, for insertion sites Ec1921, Ec2066, Ec2449 and Ec2563. The *Naegleria *sp. AM167886 sequence was used as an outgroup. Phylogenetic analysis was carried out with PAUP (version 4.0 b10; Sinauer Associates, Sunderland, MA). Gaps, encoded as missing data, and uninformative characters were excluded from the analysis. The MP trees were obtained from heuristic searches using TBR branch swapping [35], and all MP trees were within a single tree where all branch lengths equal to zero were collapsed by polytomies. A bootstrap full heuristic analysis consisting of 1000 replicates was performed and a 50% majority rule tree was produced.

The sequences corresponding to the introns from *M. anisopliae *obtained in this study were deposited in the GeneBank database under the accession numbers given in Table 1.

## Authors' contributions

MM carried out laboratory work, participated in sequence alignments, and wrote the draft of manuscript. EAI reviewed the laboratory work. EQM and CSA provided the *M. anisopliae *isolates. In addition, EQM participated in genomic DNA extraction. EM conceived the design of the study and helped to review the manuscript. RH participated in the design and coordination of the study, carried out sequence phylogenetic analyses and helped to write the manuscript. All authors have read and approved the final manuscript.

## Figures and Tables

**Table 1:** Source and origin of the *Metarhizium anisopliae *isolates used, and intron sequence accession numbers obtained in this study.

**Code**	**Source**	**Location**	**Habitat/Host**	**GeneBank No.**			
				**Ec1921**	**Ec2066**	**Ec2449**	**Ec2563**
Ma1	EAMa 00/09-Su	Cabeza de Buey, Badajoz	mmeadow	DQ667583		DQ667589	DQ667591
Ma2	EAMa 00/25-Su	Laguneta, Tenerife	meadow			DQ667587	
Ma3	EAMa 01/16-Su	Velez Rubio, Almería	wheat	DQ667583		DQ667586	
Ma4	EAMa 01/42-Su	El Carpio, Córdoba	wheat				
Ma5	EAMa 01/54-Su	Carchelejo, Jaén	scrubland	DQ667583		DQ667587	
Ma6	EAMa 01/94Su	Darro, Granada	sunflower	DQ667583		DQ667588	DQ667592
Ma7	EAMa 01/97-Su	Setubal (Portugal)	littoral				
Ma8	EAMa 01/106-Su	Almadén de la Plata, Sevilla	holm oak	DQ667583		DQ667589	DQ667591
Ma9	EAMa 01/131-Su	La Barca de la Frontera, Cádiz	pine	DQ667583		DQ667589	DQ667591
Ma10	EAMa 01/138-Su	Trebujena, Cádiz	sunflower	DQ667583		DQ667589	DQ667591
Ma11	EAMa 01/144-Su	Osuna, Sevilla	cotton	DQ667583		DQ667589	DQ667591
Ma12	EAMa 01/149-Su	Fuente de Piedra, Málaga	cotton			DQ667590	DQ667593
Ma13	EAMa 01/161-Su	Valverde del Camino, Huelva	holm oak				
Ma14	EAMa 92/9-Dm	Badajoz	*Dociostaurus maroccanus*	DQ667583		DQ667589	DQ667591
Ma15	EAMa 04/01-Ci	Tbilisi (Greorgia)	*Calliptamus italicus*	DQ667583		DQ667589	DQ667591
Ma16	EAMa 01/44-Su	Jidar, Jaén	scrubland	DQ667583		DQ667589	DQ667591
Ma17	EAMa 01/121-Su	Marchena, Sevilla	cotton		DQ667585		
Ma18	EAMa 01/58-Su	Hinojosa del Duque, Córdoba	wheat				
Ma19	EAMa 04/03-Su	San Vicente Toranzo, Cantabria	corn				
Ma20	EAMa 04/04-Su	Puerto Palombera, Cantabria	grassland				
Ma21	EAMa 04/05-Su	San Felices de Buelna, Cantabria	grassland	DQ667584			
Ma22	EAMa 04/06-Su	Valle Cabuerniga, Cantabria	grassland	DQ667584			
Ma23	EAMa 04/07-Su	Fontane de Ruente, Cantabria	grassland				
Ma24	EAMa 04/09-Su	Marañon, Navarra	wheat				
Ma25	EAMa 04/10-Su	Marquínez, Álava	potato				
Ma26	EAMa 04/02-Su	Argamasilla de Alba, Ciudad Real	vine	DQ667583		DQ667589	DQ667591

Code: reference as every isolate is cited in the text and figure 1.

Source: reference as received from the Collection of Ciencias y Recursos Agrícolas y Forestales (CRAF) Department, University of Cordoba, Spain.

Location: Spanish sites where the isolates were obtained. All isolates were from Spain, except Ma7, from Portugal, and Ma15, from Georgia.

GeneBanK No.: accession numbers of the intron LSU rDNA sequences in the GeneBank database. Ec1921, Ec2066, Ec2449 and Ec2563 represent the insertion sites. Identical intron sequences have the same accession number.

**Table 2:** Genotypes derived from the presence/absence of group I introns and their distribution frequencies in the twenty-six *Metarhizium anisopliae *isolates

**No**	Genotype*	**Isolates**	**No. isolates**	**Frequency (%)**
1	A1 B2 A3 A4	Ma1, Ma6 Ma8–11, Ma14–16, Ma26	10	38.5
2	B1 B2 A3 A4	Ma12	1	3.8
3	A1 B2 A3 B4	Ma3 and Ma5	2	7.7
4	B1 B2 A3 B4	Ma2	1	3.8
5	B1 A2 B3 B4	Ma17	1	3.8
6	A1 B2 B3 B4	Ma21–22	2	7.7
7	B1 B2 B3 B4	Ma4, Ma7, Ma13, Ma18–20, Ma23–25	9	34.6
		Total	26	100

*A, presence, and B, absence of a given intron within the 3' -end of the nuclear LSU rDNA genes. Numbers 1–4 represent the insertion sites Ec1921, Ec2066, Ec2449 and Ec2563, respectively.
